# Physical Properties and Hydration Characteristics of Low-Heat Portland Cement at High-Altitude

**DOI:** 10.3390/ma16083110

**Published:** 2023-04-14

**Authors:** Ning Wang, Qiang Liu, Yanqing Xia, Jun Li, Zhongyuan Lu, Yigang Xu, Wen Zhong, Yan Lin

**Affiliations:** 1 State Key Laboratory of Environment-Friendly Energy Materials, Department of Materials, Southwest University of Science and Technology, Mianyang 621010, China; 2 Jiahua Special Cement Co., Ltd., Leshan 614003, China

**Keywords:** low-heat Portland cement (PLH), high-altitude environment, strength, drying shrinkage, hydration characteristics, pore, Ca/Si ratio

## Abstract

High-altitude environments are characterized by low air pressures and temperature variations. Low-heat Portland cement (PLH) is a more energy-efficient alternative to ordinary Portland cement (OPC); however, the hydration properties of PLH at high altitudes have not been previously investigated. Therefore, in this study, the mechanical strengths and levels of the drying shrinkage of PLH mortars under standard, low-air-pressure (LP), and low-air-pressure and variable-temperature (LPT) conditions were evaluated and compared. In addition, the hydration characteristics, pore size distributions, and C-S-H Ca/Si ratio of the PLH pastes under different curing conditions were explored using X-ray diffraction (XRD), thermogravimetric analysis (TG), scanning electron microscopy (SEM), and mercury intrusion porosimetry (MIP). Compared with that of the PLH mortar cured under the standard conditions, the compressive strength of the PLH mortar cured under the LPT conditions was higher at an early curing stage but lower at a later curing stage. In addition, drying shrinkage under the LPT conditions developed rapidly at an early stage but slowly at a later stage. Moreover, the characteristic peaks of ettringite (AFt) were not observed in the XRD pattern after curing for 28 d, and AFt transformed into AFm under the LPT conditions. The pore size distribution characteristics of the specimens cured under the LPT conditions deteriorated, which was related to water evaporation and micro-crack formation at low air pressures. The low pressure hindered the reaction between belite and water, which contributed to a significant change in the C-S-H Ca/Si ratio in the early curing stage in the LPT environment.

## 1. Introduction

Currently, concrete is a widely used building material, and Portland cement (PC) is a commonly used cementitious material, with a global output of approximately 4 billion metric tons [1]. The construction, operation, and maintenance of the building sector consume approximately half of the annual global energy supply [2,3], and China accounts for >57% of global PC production and consumption [4]. Considering the proposed carbon reduction measures in China, the use of low-energy-consumption PC to replace ordinary PC (OPC) in building construction is an effective method of reducing energy consumption [5,6].

Low-heat PC, hereafter denoted PLH, contains a high belite content [7], i.e.: C_2_S is the main mineral component of PLH, with a content of >40% [8]. The theoretical energy consumption in preparing 1 kg C_2_S is 1350 kJ, which is 460 kJ lower than that of 1 kg C_3_S [9,10]. Therefore, the burning temperature of PLH clinker is 1250–1350 °C, which is 100 °C lower than that of OPC [11,12,13]. C_2_S reacts slowly with water [14] but displays good hydraulic properties [15]. In terms of the mechanical properties of the mineral components of the cement, the minerals are ranked in the following order of decreasing performance: C_3_S > C_4_AF > C_3_A > C_2_S, and the order of heat release for hydration is C_3_A > C_3_S > C_4_AF > C_2_S [16]. Therefore, the main hydration characteristics of PLH are its low hydration heat and delay in attaining the hydration heat maximum. The morphology of PLH is denser than that of OPC, and it exhibits a favorable compressive strength [17,18]. Owing to its low heat release in the early stage, PLH may effectively reduce cracks caused by thermal stress in the early stage of mass concrete, reduce early water migration, and decrease the drying shrinkage of concrete. As the early hydration heat release of PLH is small, the increase in the temperature of the mass concrete may be controlled, and the formation of temperature cracks may be reduced. Therefore, PLH is often used in the construction of mass concrete projects, such as hydropower stations [19].

Owing to the rapid development of western China, infrastructure construction in high-altitude areas is increasing. However, the distinct environmental conditions [20] (low air pressure and large temperature variation) in high-altitude areas significantly affect the hydration characteristics of OPC and reduce the compressive strength and durability of concrete [21,22], thereby affecting the engineering life.

Environments with low air pressures and large temperature variations adversely affect the early hydration and physical properties of OPC [23,24,25,26]. However, the hydration characteristics of PLH in these environments have not been previously reported. The effects of low air pressures and temperature variations on the mechanical strength, drying shrinkage, hydration characteristics, pore size distribution, and C-S-H Ca/Si ratio of PLH were investigated to provide a basis for its application in high-altitude environments.

## 2. Materials and Experimental Methods

### 2.1. Raw Materials

**Figure 1:**
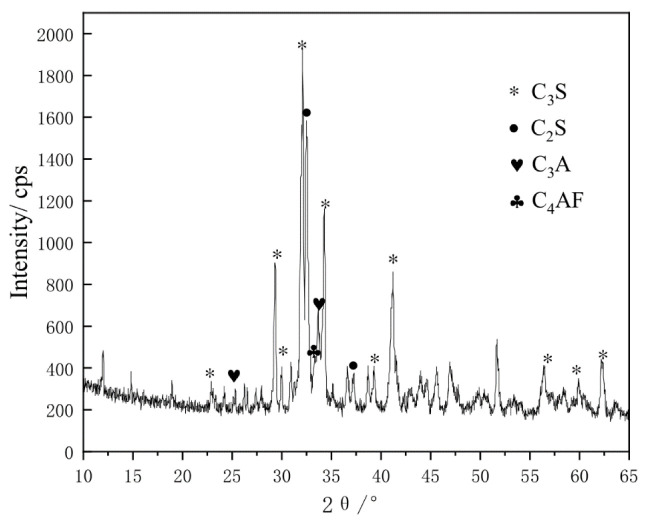
XRD pattern of PLH.

In this study, PLH42.5 was supplied by Jiahua Special Cement, Leshan, China, and its chemical composition is listed in Table 1. The mineralogical composition of PLH42.5 is listed in Table 2, and its X-ray diffraction (XRD) pattern is shown in Figure 1.

### 2.2. Sample Preparation and Maintenance

#### 2.2.1. Sample Preparation

Mixtures comprising PLH, sand, and water in a mass ratio of 1:3:0.5 were mixed using a mechanical stirrer and poured into molds with dimensions of 40 × 40 × 160 mm. The fresh PLH mortars were cured for 1 d at 20 °C and 90% relative humidity (RH) and demolded, and thereafter, the curing was continued as described in Section 2.2.2, until the evaluation age of the mechanical strength.

According to the Chinese Standard JC/T 603-2004 [27], prism specimens with sizes of 25 × 25 × 280 mm were prepared for use while evaluating drying shrinkage under high-altitude conditions. These specimens were demolded after curing for 1 d at 20 °C and 90% RH; thereafter, the curing was continued as described in Section 2.2.2.

In addition, pastes comprising PLH42.5 and water in a mass ratio of 1:0.5 were poured into molds (20 × 20 × 20 mm). These specimens were demolded after curing for 1 d at 20 °C and 90% RH, and thereafter, the curing was continued as described in Section 2.2.2, until the evaluation age for characterization using thermogravimetric analysis (TG), X-ray diffraction (XRD),mercury intrusion porosimetry (MIP), scanning electron microscopy (SEM), and energy-dispersive X-ray spectroscopy (EDS).

The details of the specimens used in the different experiments are listed in Table 3.

#### 2.2.2. Curing Regimes

The 40 × 40 × 160 mm and 20 × 20 × 20 mm specimens described in Section 2.2.1 were divided into three groups and cured under three conditions for 3, 7, 28, or 90 d, respectively. The three groups are denoted PLH42.5, PLH42.5-LP, and PLH42.5-LPT. The first condition was the standard condition (20 ± 1 °C, 90% RH, 101 kPa), and the second and third conditions simulated the characteristics of high-altitude areas. A customized environmental maintenance instrument was used to provide the respective pressure and temperature adjustments of 100–0 kPa and –45 to 80 °C, and the specimens of the second and third groups were cured using this instrument. The second condition was the low-air-pressure condition (20 ± 1 °C, 10% RH, 50 kPa), and the third condition was the condition with a low air pressure and temperature variation (5–60 °C, 10% RH, 50 kPa). The process of temperature variation was as follows: heating and cooling for 2 h, constant temperature for 20 h, and a temperature cycle of 24 h. In addition, to adopt moisturizing curing measures closer to those of the aging in the project, high-humidity curing (90% RH) was adopted up to 7 d of aging under the second and third curing conditions, and low-humidity curing (10% RH) was adopted after 7 d. The other parameters remained unchanged.

The specimens with dimensions of 25 × 25 × 280 mm were cured in water for 2d and measured the initial length, then cured under the following conditions: standard (20 ± 1 °C, 50% RH, 101 kPa), low air pressure and humidity (20 ± 1 °C, 10% RH, 50 kPa), or low pressure and humidity with temperature variation (5–60 °C, 10% RH, 50 kPa). The dry shrinkage rate was measured at different ages after exposure to three different environments.

The curing regimes of the specimens are listed in Table 4.

### 2.3. Test Method

#### 2.3.1. XRF

The chemical compositions of the raw materials were determined using X-ray fluorescence analysis (Axios X, Malvern Panalytical, Malvern, UK).

#### 2.3.2. Mechanical Strength

The flexural and compressive strengths of the mortars were evaluated after curing for 3, 7, 28, or 90 d under different conditions. The mechanical performance was measured according to ISO 679-2009 [28]. Three mortar specimens were evaluated at the desired ages using a SANS CMT5105 (MTS, Shanghai, China) to determine the average flexural and compressive strengths.

#### 2.3.3. Dry Shrinkage

The degrees of the drying shrinkage of mortar prisms with dimensions of 25 × 25 × 280 mm were investigated according to ASTM C596-2009 [29] (USA) and JC/T 603-2004 (China). The initial lengths of the specimens were measured before curing in different environments, and the levels of drying shrinkage were calculated based on the changes in the lengths of the specimens. Three specimens were evaluated for each curing condition to determine the average degrees of drying shrinkage.

#### 2.3.4. XRD-Rietveld Analysis

The mineral compositions of the raw material and hardened pastes were determined using XRD (D8 ADVANCE, Bruker, Billerica, MA, USA) with Cu Kα radiation (λ = 1.5406 Å) at 60 kV and 80 mA. The powders were scanned at 2θ values from 5° to 70° with a step size and time per step of 0.02° and 0.5 s, respectively. The mineral composition of PLH was determined via analysis using the Bruker EVA V4.2.0.14 search/match software. Quantitative phase analysis via Rietveld refinement was performed using the Bruker TOPAS 5.0 software based on quantitative XRD.

#### 2.3.5. Thermal Analysis

The mass loss of the hardened paste was measured using TGA (STA 449C, NETZSCH, Selb, Germany) between 30 and 1000 °C at a heating rate of 20 °C/min in a N_2_ atmosphere.

#### 2.3.6. SEM and EDS

The morphologies of the specimens and elemental compositions of the hydrated products formed under the three conditions were investigated using SEM–EDS (Ultra 55, ZEISS, Oberkochen, Germany).

#### 2.3.7. Pore Structure

The pore size distributions of the hardened specimens were studied using MIP with Hg intrusion (PoreMaster 60GT, Quantachrome, Boynton Beach, FL, USA).

## 3. Result and Discussion

### 3.1. Mechanical Strength

**Figure 2:**
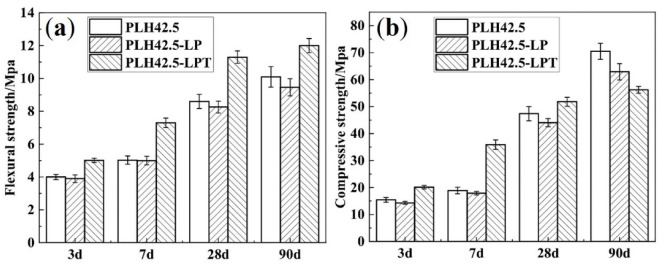
(**a**) Flexural and (**b**) compressive strengths of the mortars.

The mechanical performances of the mortars cured under different conditions are shown in Figure 2. Figure 2a,b shows the respective flexural and compressive strengths of the mortars cured under three different conditions. The strength of the mortar cured in the LP environment is slightly lower than that cured under standard conditions at all curing ages. The flexural strengths of PLH42.5-LP after curing for 3, 7, 28, and 90 d are 0.10, 0.03, 0.34, and 0.64 MPa lower than those of PLH42.5, respectively. The compressive strengths of PLH42.5-LP after curing for 3, 7, 28, and 90 d are 1.13, 0.99, 3.32, and 7.60 MPa lower than those of PLH42.5, respectively. A low-pressure environment accelerates water evaporation [30] and hinders cement hydration [23]. Simultaneously, moisture evaporation increases the drying shrinkage rates of the specimens [31], resulting in an increased number of micro-cracks in the matrices, which adversely affect the mechanical strengths [32,33].

The flexural strengths of PLH42.5-LPT after curing for 3, 7, 28, and 90 d are 4.1, 7.3, 11.3, and 12.0 MPa, respectively. The compressive strengths of PLH42.5-LPT after curing for 3, 7, 28, and 90 d are 15.0, 35.9, 51.8, and 56.2 MPa, respectively. The compressive strength of PLH42.5-LPT is higher than those of PLH42.5 and PLH42.5-LP before a curing age of 28 d but lower than those of PLH42.5 and PLH42.5-LP at a curing age of 90 d. This is because the hydration of belite may be enhanced at higher hydration temperatures, and superior mechanical properties may be observed in the early stages [34]. However, owing to the variable temperature in the simulated high-altitude environment, numerous micro-cracks are observed in the matrix due to thermal stress, which reduces the long-term strength of the matrix.

### 3.2. Drying Shrinkage

**Figure 3:**
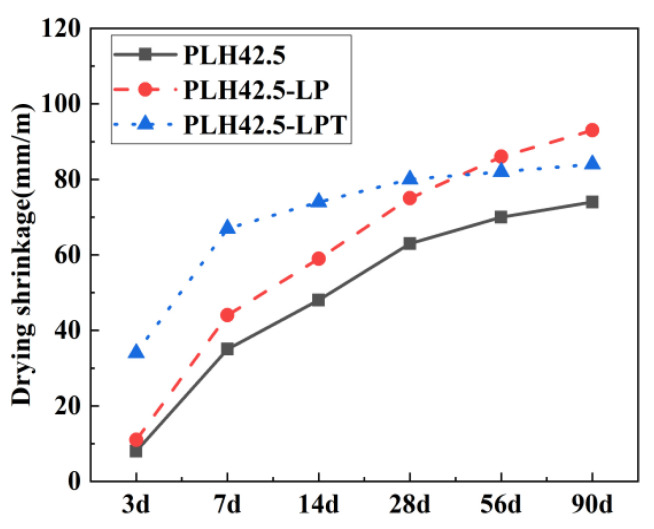
The drying shrinkage of mortars at different curing conditions.

The time-dependent levels of the drying shrinkage of the PLH mortars under different curing conditions were evaluated and compared, as shown in Figure 3. Under the standard, LP, and LPT conditions, the degrees of the drying shrinkage of the PLH mortar specimens are 74, 93, and 84 mm/m, respectively. Comparatively, the degrees of the drying shrinkage of the specimens under the LP and LPT conditions at 90 d are more than 1.26 and 1.13 times those of the specimens under standard conditions, which suggests that the low air pressure may critically accelerate drying shrinkage. This is because the low air pressure accelerates moisture evaporation, which contributes to the levels of drying shrinkage of the mortars.

Additionally, as shown in Figure 3, the drying shrinkage under LPT conditions develops rapidly at the early stage but slowly at a later stage. Under the LPT conditions, the change in the drying shrinkage is 67 mm/m before 7 d and only 17 mm/m between 28 and 90 d, i.e., 80% of the drying shrinkage over 90 d occurs in the first 7 d after mortar casting. This may be due to numerous reasons, among which two are analyzed here. First, owing to temperature variation (5–60 °C), the moisture losses of the PLH-LPT mortars in the first 7 d are consistently higher than those of the standard-cured and LP mortars. Moreover, when the mortar is cured at various temperatures, the hydration products C-S-H and Ca(OH)_2_ form rapidly, reducing the pore connectivity and water migration pathways within the PLH mortar by generating finer pore structures. Under the LPT conditions, the C_2_S in the PLH is rapidly hydrated, and the C-S-H and Ca(OH)_2_ phases fill the crystalline skeleton, improving the shrinkage resistance of the specimen.

### 3.3. Hydration Characteristics

**Figure 4:**
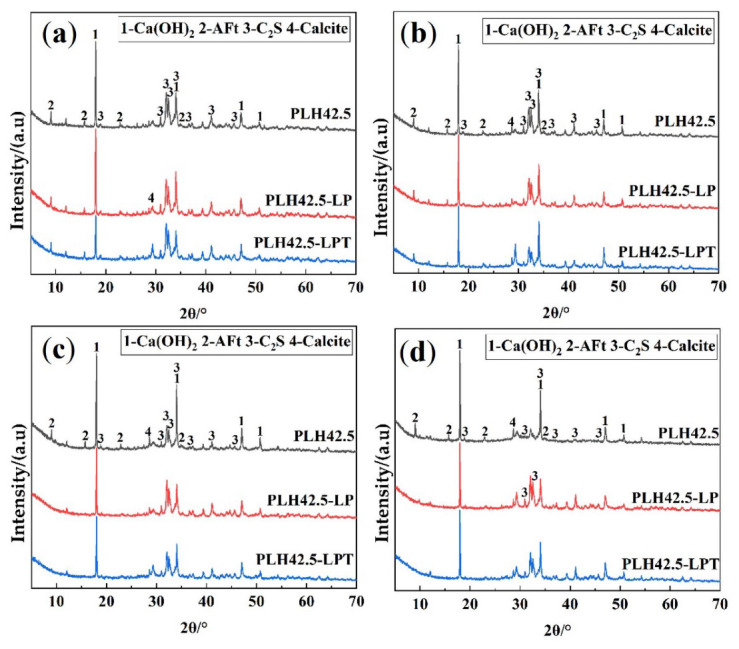
X-ray diffraction pattern of harden pastes curing for (**a**) 3 days, (**b**) 7 days, (**c**) 28 days, and (**d**) 90 days.

The XRD patterns of the hardened pastes cured under different conditions for 3, 7, 28, or 90 d are shown in Figure 4. The hydration products of PLH curing under different conditions include Ca(OH)_2_, ettringite (AFt), and calcite. The characteristic peaks of AFt are not observed in the patterns of PLH42.5-LP and PLH42.5-LPT after curing for 28 d. This is because the evaporation and migration of water are accelerated; the boiling point of water is reduced, and AFt is dehydrated to AFm at a low pressure.

**Figure 5:**
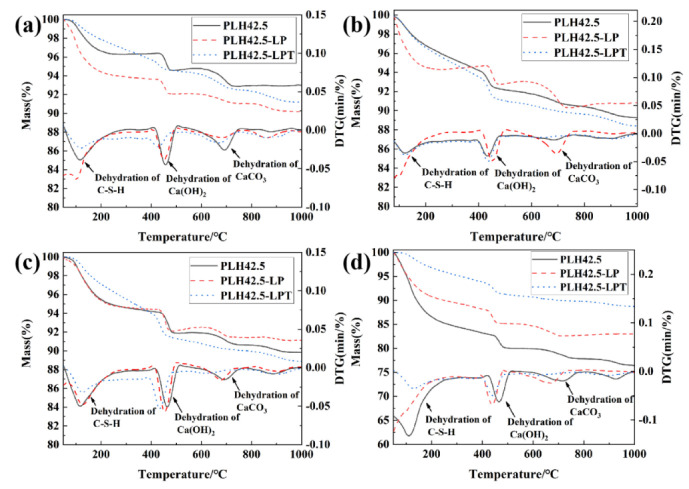
TG and DTG of the pastes cured for (**a**) 3, (**b**) 7, (**c**) 28, or (**d**) 90 d.

The results of the TG of the hardened pastes cured under different conditions after 3, 7, 28, or 90 d are shown in Figure 5. The main temperature ranges of mass loss are 100–400, 400–570, and 570–800 °C. The mass losses in these temperature ranges are indexed to the dehydration of C-S-H and AFt [35], dehydroxylation of Ca(OH)_2_ [36], and decarbonization of CaCO_3_ [37], respectively.

**Figure 6:**
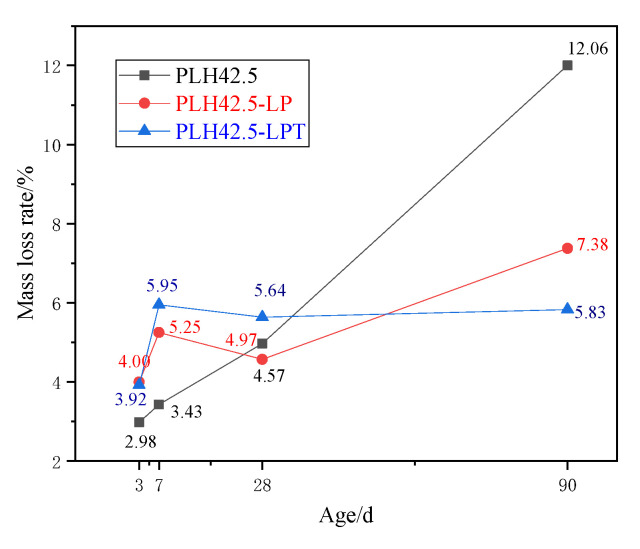
Mass loss rate of the cured pastes in the temperature range 100–400 °C.

The mass loss rate of the hardened pastes in the temperature range 100–400 °C are shown in Figure 6. When the PLH paste is cured under the standard or LP conditions, the mass loss rate of the paste in the temperature range 100–400 °C generally increases as the curing age increases. However, when the PLH paste is cured under the LPT conditions, the mass loss rate of the paste in the temperature range 100–400 °C generally decreases slightly as the curing age increases. The mass loss rate of the PLH paste increases from 2.98% to 12.06% when cured under the standard conditions and from 4.00% to 7.38% when cured under the LP conditions. The mass loss rate of the PLH paste under the LPT conditions are 3.92%, 5.95%, 5.64%, and 5.83%. The changes in the mass loss rate trend of the specimens in the temperature range 100–400 °C when cured under the different conditions are consistent with the compressive strengths. The compressive strengths of the mortars cured under the LPT conditions are higher than those of the mortars cured under the standard conditions for 3, 7, and 28 d. However, the compressive strengths of the mortars cured under the LPT conditions are lower than those of the mortars cured under the standard conditions for 90 d. C-S-H and AFt form slowly at a later curing stage, which may retard the development of the compressive strength of the mortar cured under LPT conditions.

**Figure 7:**
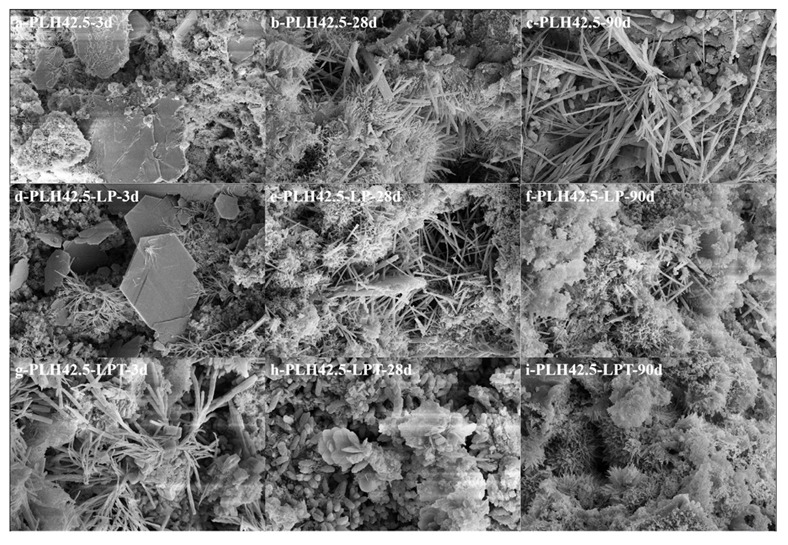
SEM image of PLH42.5 (**a**–**c**), PLH42.5-LP (**d**–**f**), PLH42.5-LPT (**g**–**i**) curing for 3 days, 28 days, and 90 days.

The SEM images of PLH42.5, PLH42.5-LP, and PLH42.5-LPT cured for 3, 28, or 90 d are shown in Figure 7. Flake-, needle-rod-, cube-, fibrous-, and gelatinous-like products are observed in the samples. The flake- and needle-rod-like hydration products are Ca(OH)_2_ and AFt [38], respectively, and the cube-, fibrous-, and gelatinous-like hydration products are calcium silicate hydrates with different Ca/Si ratios [39]. Calcium silicate hydrate exhibits different morphologies, which may be caused by water transfer and the different hydration rates of belite. This leads to a change in the Ca/Si ratio of C-S-H.

### 3.4. Pore Size Distribution

**Figure 8:**
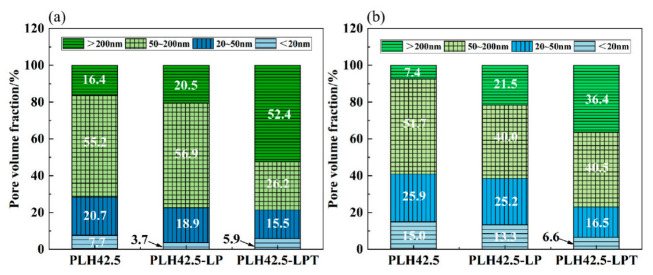
Pore volume fractions of the hardened pastes after curing for (**a**) 28 and (**b**) 90 d.

The pore volume fractions of the specimens cured for 28 and 90 d are shown in Figure 8. The pore size distribution determines the durability and physical properties of a cement-based material. As a measure of cement-based material performance, the pore size is classified as harmless (<20 nm), less harmful (20–50 nm), harmful (50–200 nm), and more harmful (>200 nm) [40]. Figure 8 shows that the pore volumes of the harmful pores (>20 nm) of the specimens increase during curing under the LP and LPT conditions compared to those of specimens cured under the standard conditions. The pore volumes of harmful pores in PLH42.5, PLH42.5-LP, and PLH42.5-LPT after curing for 28 d are 92.3%, 96.3%, and 94.1%, respectively. With increasing curing age, the pore size distribution of PLH42.5-LP is refined. The pore volume of harmful pores in PLH42.5-LP is very different from that in PLH42.5. However, the pore volume of the harmful pores in PLH42.5-LPT is higher than those of PLH42.5 and PLH42.5-LP after curing for 28 or 90 d. The increase in the harmful pore volume is related to the acceleration of water transport and evaporation and micro-crack formation at low air pressures.

### 3.5. Ca/Si Ratio of C-S-H at an Early Curing Stage

**Figure 9:**
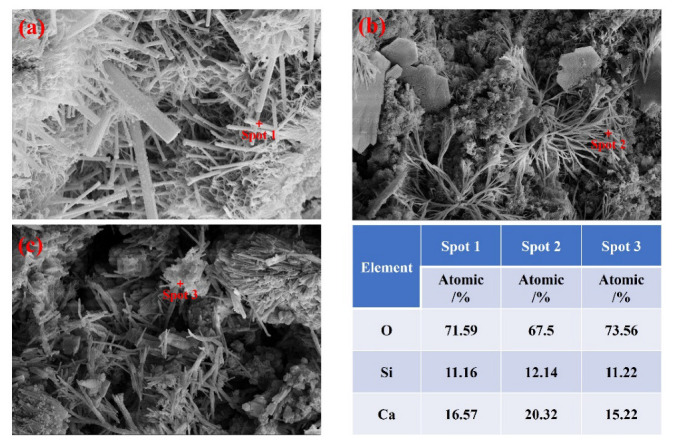
EDS of the specimens cured for 3 d: (**a**) PLH42.5, (**b**) PLH42.5-LP, and (**c**) PLH42.5-LPT.

The PLH reacts with water in a high-altitude environment with low-air-pressure conditions. This accelerates the transport and evaporation of water, which hinders the reaction between the clinker and water. This changes the hydration product morphology and CaO/SiO_2_ molar ratio (Ca/Si ratio) of C-S-H. As shown in Figure 9, significant changes in the C-S-H Ca/Si ratios are observed in different curing environments. This is because the PLH contains a large amount of belite, with a low hydration rate. When PLH is used at high altitudes, the low pressure leads to water transport and evaporation, hindering the reaction between belite and water. Figure 9a Spot 1 and Figure 9b Spot 2 indicate that low-air-pressure conditions influence belite hydration. The C-S-H Ca/Si ratio of PLH42.5 is 1.48, which is lower than that of PLH42.5-LP (1.67). Temperature is another factor influencing the hydration of belite in high-altitude environments. The temperature variation displays a positive effect on belite hydration, generating a temperature stress that leads to the formation and expansion of micro-cracks. The Ca/Si ratio of PLH42.5-LPT is 1.36 (Figure 9c Spot 3), which is lower than those of PLH42.5 and PLH42.5-LP, indicating that the hydration of PLH increases under low-air-pressure and variable temperature conditions

## 4. Conclusions

In this study, the effects of low air pressure and temperature variations on the mechanical strength, drying shrinkage, hydration characteristics, pore size distribution, and C-S-H Ca/Si ratio were investigated to provide a basis for the application of PLH in high-altitude environments. Based on the results, the following conclusions were drawn:

(1) The compressive strength of the PLH mortar cured under the LPT conditions was higher than that of the specimens cured under standard conditions at an early curing stage (3, 7, or 28 d). This was because hydration products formed faster upon curing under the LPT conditions at an early hydration stage but slower than those formed upon curing under standard conditions at a later curing stage (90 d), owing to the dehydration of the hydration products. The flexural strength was higher in the PLH mortar cured under the LPT conditions than that of the PLH cured under the standard conditions after curing for 3, 7, 28, or 90 d.
(2) A low air pressure accelerated the moisture evaporation and drying shrinkage of the PLH mortar. In addition, drying shrinkage under LPT conditions developed rapidly at an early stage but slowly at a later stage. Owing to the temperature variation (5–60 °C), the moisture losses of the PLH-LPT mortars in the first 7 d were consistently higher than those of the standard-cured and LP mortars. Moreover, when the mortar was cured at various temperatures, the hydration products C-S-H and Ca(OH)_2_ formed rapidly, which reduced the pore connectivity and water migration pathways within the PLH mortar.
(3) Hydration products were formed faster than curing products under the LPT conditions at an early hydration stage. Under the LPT conditions, the characteristic peaks of AFt were not observed in the XRD pattern after curing for 28 d, and AFt transformed into AFm.
(4) The pore size distribution characteristics of the specimens cured under the LPT conditions deteriorated, owing to water evaporation and micro-crack formation at low air pressures.
(5) Low pressure led to water transport and evaporation, hindering the reaction between belite and water and contributing to significant changes in the C-S-H Ca/Si ratios in the early curing stages in different curing environments.

The results provide a basis for material selection in generating concrete materials with cracking resistances in plateau areas. PLH exhibits a low water demand, which favors the regulation of the construction performance. A low hydration heat results in lower requirements in terms of concrete cooling and insulation, and a low shrinkage decreases the risk of shrinkage cracking and maintenance costs during construction. Hydration products display dense structures, enhanced durability, and lower maintenance costs during operation. Therefore, the comprehensive benefit of PLH is significantly higher than that of OPC, and PLH exhibits a higher application potential and economic value.

## Figures and Tables

**Table 1:** Chemical composition of PLH42.5 (wt.%).

SiO_2_	Al_2_O_3_	CaO	MgO	SO_3_	Fe_2_O_3_	K_2_O	Na_2_O	Other Components	Loss
23.57	3.98	61.33	2.55	2.34	4.75	0.37	0.22	0.26	0.63

**Table 2:** Mineral composition of PLH42.5 (wt.%).

C_3_A	C_3_S	C_2_S	C_4_AF	Other Minerals
2.53	29.97	44.96	14.44	8.10

**Table 3:** Details of the specimens used in different experiments.

Experiment	Specimen Size (mm)	Number of Specimens	Evaluation Age (d)
Mechanical strength	40 × 40 × 160	36	3, 7, 28, and 90
Drying shrinkage	25 × 25 × 280	9	3, 7, 28, 56, and 90
XRD, SEM, EDS, TG, and MIP	20 × 20 × 20	36	3, 7, 28, and 90

**Table 4:** Curing regimes.

Specimen Size (mm)	Curing Condition	Environmental Parameters	Notation
40 × 40 × 160 and 20 × 20 × 20	Standard condition	20 ± 1 °C, 90% RH, 101 kPa	PLH42.5
	Low air pressure condition (LP)	1 d: 20 ± 1 °C, 90% RH, 101 kPa 2–7 d: 20 ± 1 °C, 90% RH, 50 kPa 8–90 d: 20 ± 1 °C, 10% RH, 50 kPa	PLH42.5-LP
	Low air pressure and variable temperature condition (LPT)	1 d: 20 ± 1 °C 90% RH, 101 kPa 2–7 d: 5–60 °C, 90% RH, 50 kPa 8–90 d: 5–60 °C, 10% RH, 50 kPa	PLH42.5-LPT
25 × 25 × 280	Standard condition	20 ± 1 °C, 50% RH, 101 kPa	PLH42.5
	Low air pressure condition (LP)	20 ± 1 °C, 10% RH, 50 kPa	PLH42.5-LP
	Low air pressure and variable temperature condition (LPT)	5–60 °C, 10% RH, 50 kPa	PLH42.5-LPT

## Data Availability

Data will be made available upon reasonable request.
